# Reviving the RNA World: An Insight into the Appearance of RNA Methyltransferases

**DOI:** 10.3389/fgene.2016.00099

**Published:** 2016-06-06

**Authors:** Ajay K. Rana, Serge Ankri

**Affiliations:** ^1^Division of Biology, State Forensic Science Laboratory, Ministry of Home Affairs, Government of JharkhandRanchi, India; ^2^Department of Molecular Microbiology, The Ruth and Bruce Rappaport Faculty of Medicine, Technion Israel Institute of TechnologyHaifa, Israel

**Keywords:** RNA world, RNA methylation, DNA methylation, RNA-DNA transition, evolution

## Abstract

RNA, the earliest genetic and catalytic molecule, has a relatively delicate and labile chemical structure, when compared to DNA. It is prone to be damaged by alkali, heat, nucleases, or stress conditions. One mechanism to protect RNA or DNA from damage is through site-specific methylation. Here, we propose that RNA methylation began prior to DNA methylation in the early forms of life evolving on Earth. In this article, the biochemical properties of some RNA methyltransferases (MTases), such as 2′-*O*-MTases (Rlml/RlmN), spOUT MTases and the NSun2 MTases are dissected for the insight they provide on the transition from an RNA world to our present RNA/DNA/protein world.

## Introduction

**FIGURE 1:**
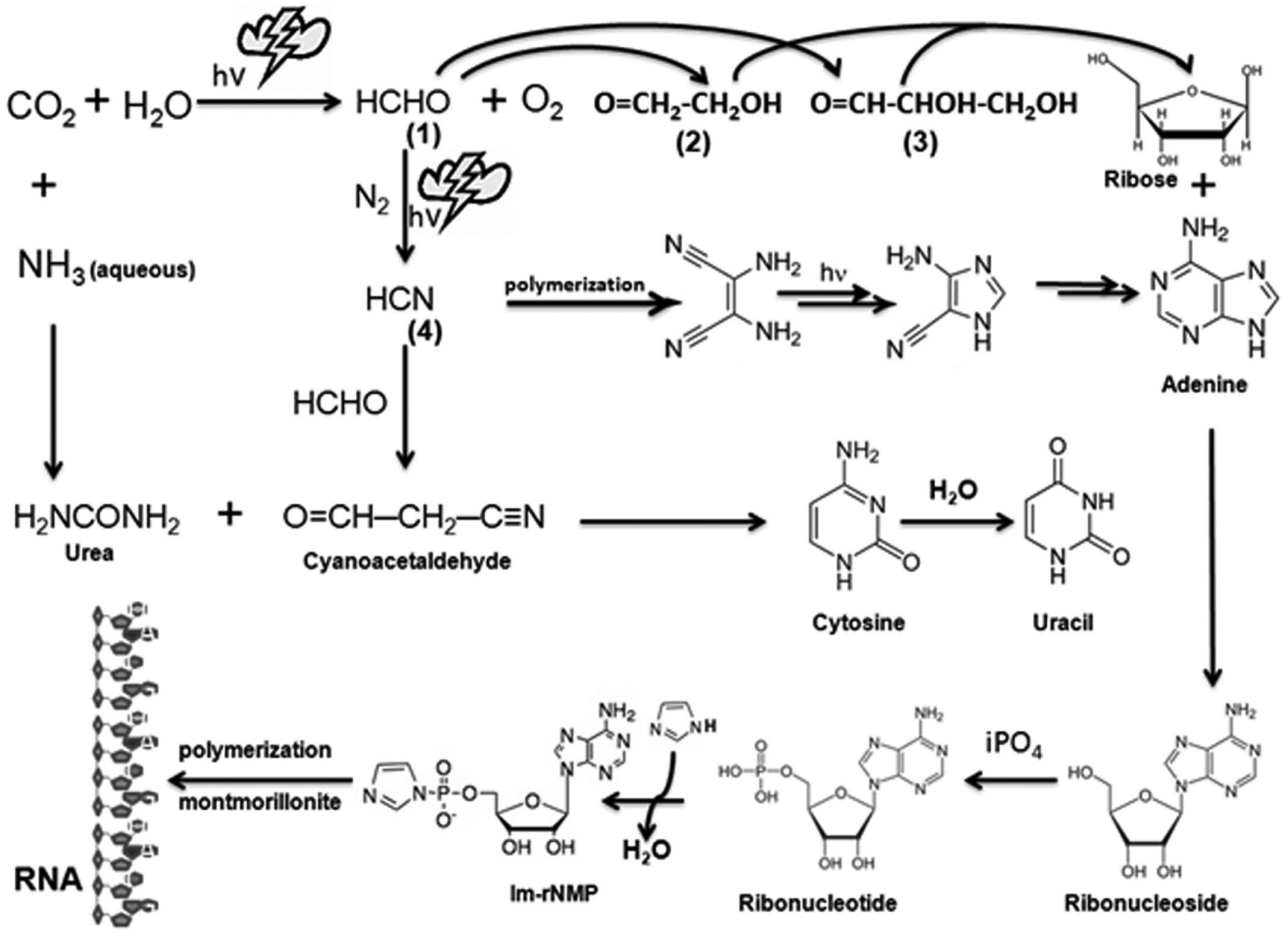
**Prebiotic synthesis of ribose, purines, pyrimidines, and RNA.** Simple inorganic molecules such as CO_2_, H_2_O, HCHO, NH_3_, and HCN, can be combined to form organic ribose sugar as well as nitrogenous bases (purines and pyrimidines) by selectively subjecting them to electrical discharges representing the proposed extreme weather conditions in the prebiotic world. The highly reactive molecule formaldehyde (1) can be generated by reacting abundant CO_2_ that was present in the reducing world with water molecules. Subsequent reaction of HCHO with itself can give rise to ribose sugar via intermediates such as glycoaldehyde (2) and glyceraldehydes (3) or another reactive molecule HCN by reacting with N_2_ under high atmospheric pressure. HCN reacts with itself to produce the purine base adenine and, with HCHO, it produces cyanoacetaldehyde, which can react with urea (H_2_NCONH_2_) to give rise to two pyrimidine bases, namely cytosine and uracil. Ribose sugar bonds with nitrogenous bases to produce ribonucleosides which might have been phosphorylated by inorganic phosphate (iPO_4_) from dissolved minerals to produce ribonucleotides (Costanzo et al., 2007). These ribonucleotides are activated by imidazole (Im) and then polymerize into a long chain without any template on a clay catalyst such as montmorillonite, which was abundantly present in the prebiotic Earth. All these steps vest on the probability of occurrence of all these ingredients and favorable conditions at least in a close proximity to the Earth surface and its proximal atmosphere. Thymine, found only in DNA, is speculated to have been synthesized with more complex reactions at a later evolutionary stage, possibly through methylation of uracil using hydrazine (H_2_NNH_2_) and HCHO.

The classic experiment performed by Miller (1953) and Miller and Urey (1959) revolutionized the view about the origin of life on Earth. In their experiment, a reducing atmosphere was generated, similar to the one proposed to have existed on the early Earth. Exposure to UV light and frequent lightning gave rise to a large number of organic molecules, some of which were precursors to the biomolecules that exist today in our biosphere (Miller and Schlesinger, 1983). These simple molecules, such as formaldehyde (HCHO) and hydrogen cyanide (HCN), might have served as the building blocks for more complex molecules such as RNA (Schrader, 2009). In the evaporating lagoons or dry beaches of early Earth, concentrated urea might have been produced which would have reacted with cyanoacetaldehyde to form cytosine; further hydrolysis of cytosine can yield another base uracil (Robertson and Miller, 1995). The ribose sugar can be formed through polymerization of HCHO itself by the formose reaction (Shapiro, 1988). Moreover, urazole, a heterocyclic compound which is isosteric with uracil’s hydrogen-bonding segment reacts spontaneously with ribose (and other aldoses) to form a mixture of four ribosides: alpha (α) and beta (β) pyranosides and furanosides (Kolb et al., 1994). The entire process could be chemically or photochemically driven, forming the basic units of ribonucleotides which, on polymerization with the help of a catalyst such as montmorillonite (Ferris and Ertem, 1993), converted free ribonucleotides into a long chain of RNA (**Figure 1**). Nature’s selection of contemporary bases in RNA might have been driven by modifications like *N*-glycosyl bonds that made the bases more resistant to hydrolysis (Rios et al., 2014). The hallmark complexity of extant forms of life may trace their origin to the catalytic RNA and the appearance of methionine and other amino acids synthesized in the highly reactive primordial soup (Van Trump and Miller, 1972; Keefe et al., 1995; Parker et al., 2011; Shechner and Bartel, 2011). Nucleophilic attack of RNA by its 2′-OH group as a nucleophile on other organic molecules, including amino acids and short peptides, was extremely useful to form RNA-protein complexes, which may represent the precursors of ribosomes (Jeffares et al., 1995; Poole et al., 1998; Melendez-Hevia, 2009). The flexibility of single-stranded RNA may have allowed it to assume various 2 and 3° structures that could form a catalytic pocket such as the pseudoknot structure present in the triple helix of telomerase (Mihalusova et al., 2011). Indeed, the mechanism used by RNA molecules to catalyze phosphodiester bond formation and cleavage using two essential magnesium ions is similar to that employed by RNA polymerases (Steitz and Steitz, 1993; Jeruzalmi and Steitz, 1998). The ability of RNA to act as a genetic material as well as an enzyme driving peptide synthesis using 23S rRNA (Tamura and Schimmel, 2001; Sun et al., 2002; Cui et al., 2004; Orgel, 2004) forms the basis for the postulated “RNA world" (Hirao and Ellington, 1995; Orgel, 2004; Muller, 2006; Manrubia and Briones, 2007). Accumulation of various RNAs (Ekland and Bartel, 1996; Orgel, 2004; Shechner and Bartel, 2011), ribosomes (Melendez-Hevia, 2009; Harish and Caetano-Anolles, 2012), RNA helicases, codon-based protein synthesis (Zenkin, 2012) and DNA, along with other organic macromolecules such as carbohydrates and lipids that assembled as coacervate droplets, may have constituted the first basic cellular structure that appeared approximately 3 billion years ago (Cooper, 2000).

**FIGURE 2:**
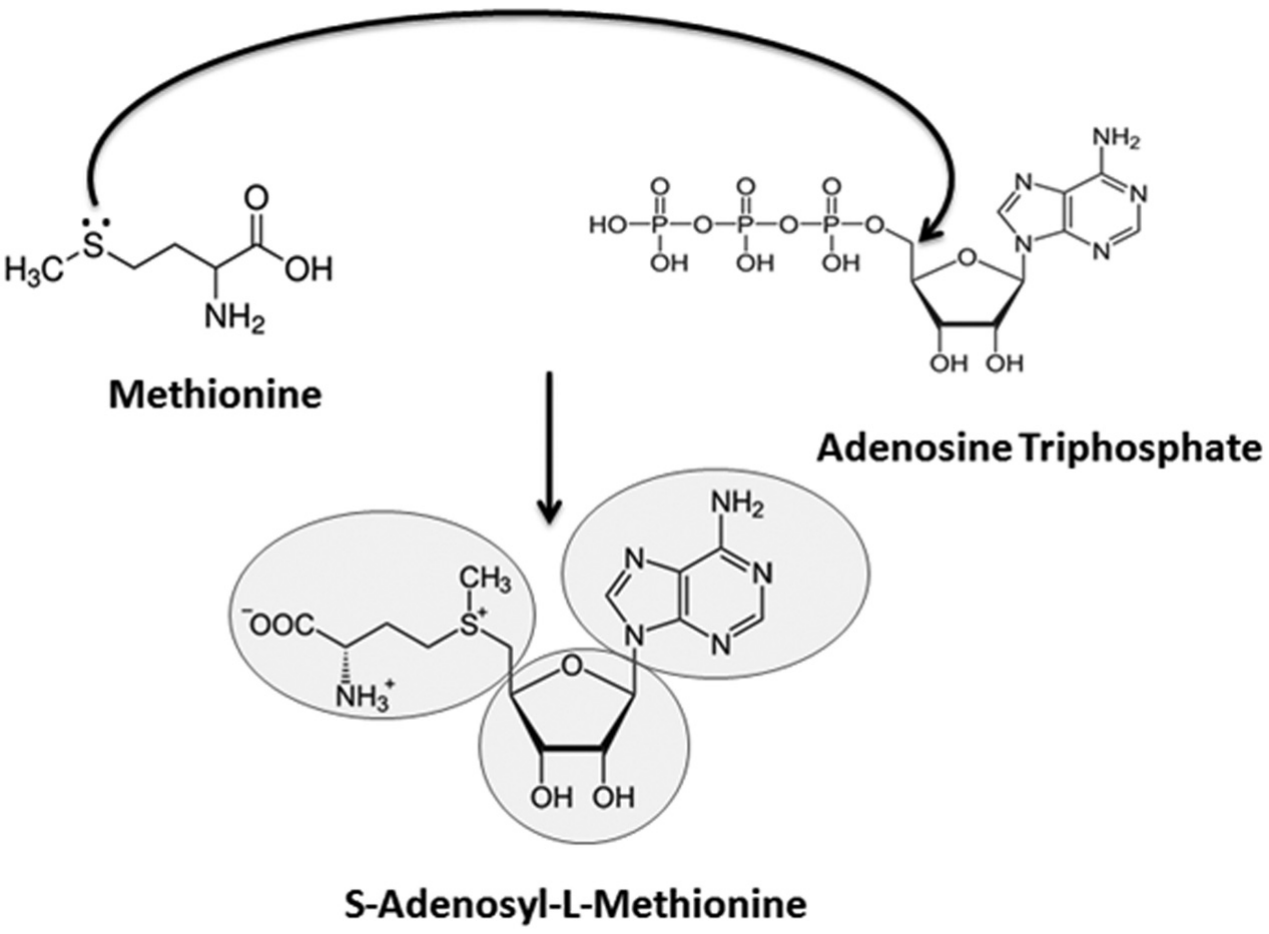
**Possible route of *S*-adenosyl-L-methionine (SAM) appearance and its metabolic significance.** The nitrogenous nucleobase ‘adenine,’ present in the universal methyl donor cofactor SAM, could have been generated by an electric spark reaction in an aqueous solution of NH_3_ and HCN (in fact, adenine is merely a pentamer of HCN). The base is highly conspicuous in all life forms in the form of the high energy molecule, Adenosine Triphosphate (ATP), which is the universal energy currency of cells. Likewise, methionine has been reported to be synthesized from a mixture of CH_4_, H_2_S, NH_3_, and CO_2_ by providing a similar electric discharge (Van Trump and Miller, 1972; Parker et al., 2011). Methionine is the first amino acid decoded by the genetic code into the biotic proteins. Finally, the ribose sugar present in SAM can be synthesized from formaldehyde by formose reaction using basic substances, neutral clays, heat and various types of radiation. Ribose is the first sugar formed in the anabolic reactions while deoxyribose is synthesized later. Nucleophilic addition reaction of methionine with ATP produces the SAM cofactor, which represents the second most abundant molecule (after ATP) inside all cells and participates in all methylation reactions.

The prebiotic appearance of RNA has received considerable attention during the past decade and this event has been succinctly summarized by Pressman et al. (2015). The chemical instability of RNA leads some researchers to argue against the concept of the RNA world (Miller and Lazcano, 1995; Levy and Miller, 1998). Other researchers propose that the RNA stability issue is exaggerated (Meyers et al., 2004) and that this problem is compensated for by some unique properties of RNA, including self-replication (Johnston et al., 2001; Zaher and Unrau, 2007; Attwater et al., 2013) and the direct involvement of RNA in peptide synthesis (Schimmel and Henderson, 1994; Tamura and Schimmel, 2003). Consequently, it has been proposed that RNA, which can serve both as genetic material and as an efficient catalyst, can support a minimal form of life (Flores et al., 2004; Koonin and Dolja, 2014). However, RNA is vulnerable to highly acidic (pH < 3) and highly basic (pH > 7) microenvironments; and sometimes to RNA itself, as in the case of self-cleaving ribozyme RNA (Long and Uhlenbeck, 1993; Jayasena and Gold, 1997; Abouhaidar and Ivanov, 1999; Kikovska et al., 2005; Ferre-D’Amare and Scott, 2010). The protection of early RNA molecules in the extreme environment of early Earth was a prerequisite for the formation of higher-order RNA macromolecules and for the development of complex forms of life (Paolella et al., 1992). Small RNA-mediated RNA methylation could represent one of the earliest mechanisms that appeared in the early RNA world to protect RNA from hydrolytic attack (Usher and McHale, 1976; Clouet d’Orval et al., 2001). Later, with the evolution of replicating and peptide-coding abilities in RNA molecules, the universal genetic code of protein synthesis began to appear, and proteins like RNA MTases assumed the responsibility for methylation activity, taking over from the catalytic RNAs (Poole et al., 1998). In the history of nucleic acid evolution, a remarkable enzyme called ribonucleotide reductase solved the problem of RNA vulnerability by converting ribose sugar to deoxyribose (Reichard, 1993). DNA began to proliferate rampantly and was selected by nature as a more stable genetic molecule than RNA for sustaining and organizing life (Melendez-Hevia, 2009). However, the appearance of DNA cannot be traced back far further than the appearance of RNA since the synthesis of the sole unique base of DNA (thymine) is carried out by a unique methyltransferase (thymidylate synthase) which requires the use of 5,10-methylenetetrahydrofolate (Mishanina et al., 2012). This observation suggests that the appearance of DNA occurred before the apparition of canonical MTases which use *S*-adenosyl-L-methionine (SAM) as a cofactor. In fact, folate is required for synthesis of SAM and as a cofactor to speed up the methylation reaction (Duthie et al., 2002). Perhaps, the selection of SAM over folate and its primordial synthesis have a different history to unfold (**Figures 1** and **2**) since its precursors are derived from adenine (the first base likely to have been synthesized in the prebiotic soup), methionine (the first amino acid added during translation) and ribose (the first sugar made in nucleic acid anabolism).

The current view about the emergence and early evolution of life is that there might have been a systematic, cooperative and coherent evolution of RNA and DNA, including the genetic code, enzymes, cofactors and methylation machinery, around 3 billion years ago, somewhere on Earth where one event was supported by the other (Higgs and Lehman, 2015). A large number of discoveries and evolving data (ribonucleotide reductase, reverse transcriptase, self-splicing RNA, catalytic RNA etc.) strongly support RNA as the first genetic material and DNA as the later in the evolutionary genealogy. However, how the predominance of DNA over RNA occurred is still an enigma and a poorly described topic. The peculiar perspective that evolution of DNA from RNA through a 2′-*O*-methyl intermediate has already been pointed in earlier literature (Jeffares et al., 1995; Poole et al., 1998, 2000) but has been a neglected topic of discussion thereafter. We have here for the first time composed a brief review of the appearance of RNA polymers and an analysis of various MTases, stability factors for RNA through methylation and the evolutionarily transition of RNA to DNA (with consequent shifting of RNA-MTases’ specificity to DNA-MTases). In summary, we present here some evolutionary evidence which supports the likelihood that RNA methylation activity began prior to DNA methylation and that 2′-*O*-methylation was probably the form of primitive methylation on RNA, which eventually led to the emergence of DNA, a molecule that ultimately shaped the form of life on Earth.

## Ribose 2′-*O*-Methylation

The transition from RNA to DNA appears to require intermediate steps, and it has been suggested that the naturally occurring 2′-*O*-methylated RNA, which has chemical properties intermediate to RNA and DNA, is a suitable candidate (Poole et al., 2000). Ribose 2′-*O*-methylation occurs in rRNA, tRNA, mRNA, snoRNA, and siRNA etc. at adenosine, guanosine, cytidine, and uridine nucleobases (Al-Arif and Sporn, 1972) and is ubiquitous in viruses, archaebacteria, eubacteria, yeasts, protists, fungi, and higher eukaryotes (Feder et al., 2003). 2′-*O*-methylation of RNA by other RNA molecules (ribozyme) or RNA complexes is indeed the primordial manifestation of the methyl transfer reaction and is the most likely mechanistic platform for the *de novo* creation of deoxyribose sugar (and, in fact, the creation of the DNA molecule) on Earth before the appearance of genetic code or ribonucleotide reductase. In fact, 2′-*O*-methylation or the insertion of an oligodeoxynucleotide (short piece of DNA) in some ribozymes leads to enhanced activity (Goodchild, 1992). Furthermore, the abstraction of the 2′-OCH_3_ moiety requires less energy than the abstraction of a hydroxyl group at the 2′ position, thus allowing the conversion of ribose to deoxyribose in a more energetically favorable manner. Two mechanisms of ribose 2′-*O*-methylation are known: one is via a site-specific 2′-*O*-MTase that belongs to the spoUT family of RNA-MTases (discussed below) and the other is through C/D box ribonucleoproteins (RNPs; Clouet d’Orval et al., 2001; Clouet-d’Orval et al., 2005). The latter includes snoRNAs that select particular sites of an RNA substrate using its C/D box and acts as a chaperone role rather than as a ribozyme to methylate its substrate. Remarkably, these functions are very similar to the functions presumably exhibited by ancient RNA.

## MTases with Dual RNA-Substrate Specificity

**FIGURE 3:**
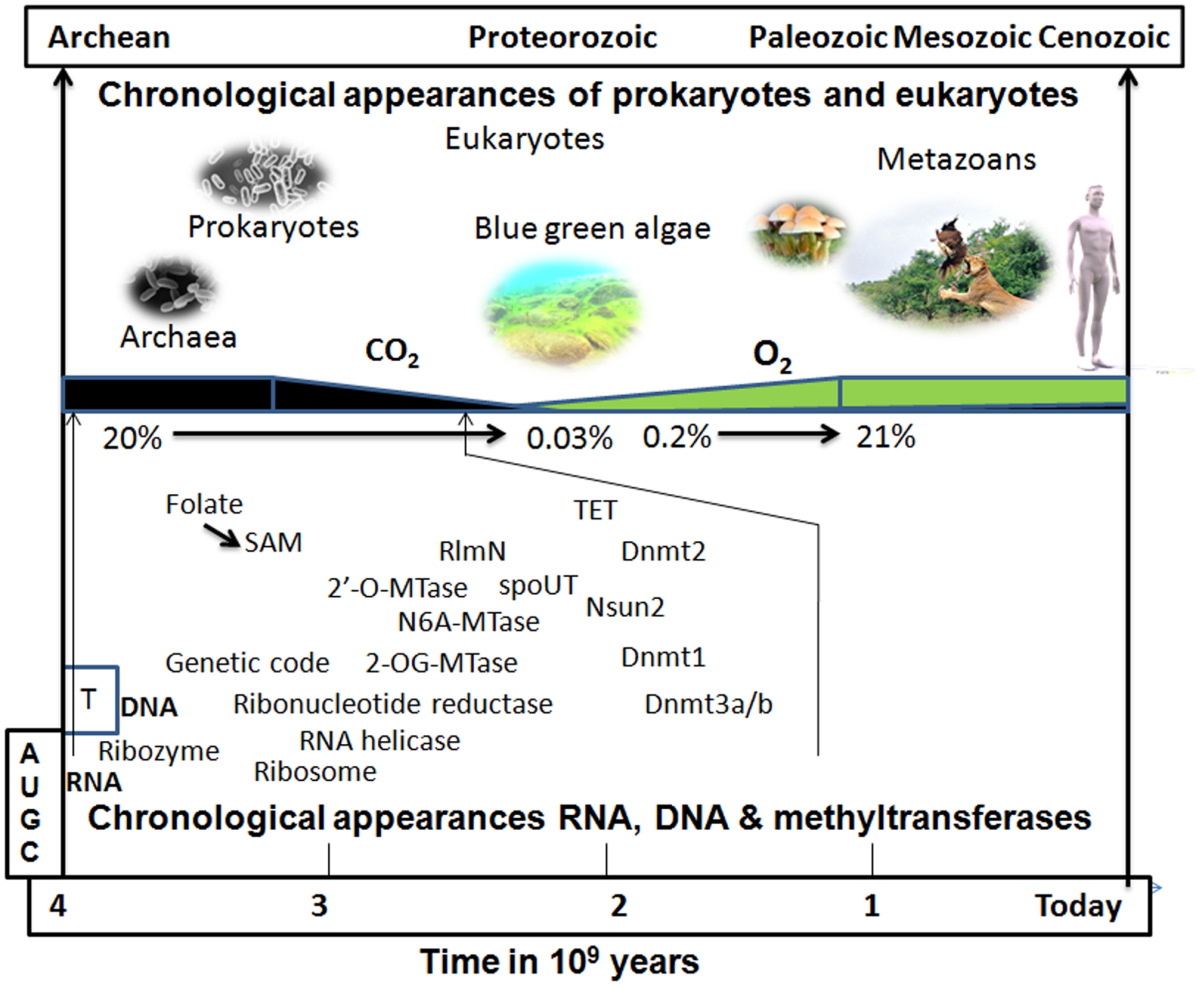
**Putative chronological appearance of RNA, DNA, MTases, prokaryotes, and eukaryotes.** RNA, which functions as a genetic material, as a chaperone, and as a peptide synthesizer, is a likely candidate as the sole common precursor of other biomolecules *de novo* in the prebiotic world. RNA modification through snoRNA (RNA acting as a ribozyme) would have been the initial step to stabilize and protect RNA from the extreme environmental conditions of the prebiotic soup on Earth. An initial DNA-like molecule would have arisen from the non-enzymatic reduction of 2′-OH of ribose sugar rather than with ribonucleotide reductase which might have manifested after the appearance of the genetic code. The kingdom of prokaryotes and Archaea that arose on early Earth already possessed RNA, DNA, and their modifying enzymes in the course of evolution. There were many RNA MTases that were initially multi-specific, i.e., acting on many RNA species and some of them, later on, may have evolved to act on DNA. With the appearance of cyanobacteria (blue–green algae), the atmospheric CO_2_ of the reducing Earth started to be consumed and its concentration gradually decreased while the concentration of O_2_ started to build up (leading to the oxidizing atmosphere of the present-day Earth). Many enzymes shifted their mechanism of methylation away from depending on radical SAM (an anaerobic type of methylation; Zhang et al., 2011) to nucleophilic attack (S_N_2 type) and evolved to become oxygen tolerant. The presence of oxygen may have triggered the reversibility of methylation reaction since demethylases (TET1-3) are often dioxygenases (Tsukada, 2012).

The two known family of MTases involved in methylation of rRNA, as well as tRNA, are spoUT family and RlmN enzymes. The spoUT enzymes employ a minimal domain to carry out the methylation reaction, though the exact binding and catalytic mechanisms remain elusive (Liu et al., 2013). spoUT genes are abundant in extremophiles (pyrococcus, methanococcus, acidolobus, thermophilus, metalophilus, and halophilus)^1^. These enzymes might be involved in the control of ribosome or tRNA activity and in the regulation of protein synthesis during the cellular stress response. The other family of RNA-MTase (RlmN) has been reported to methylate both rRNA and tRNA at the second aromatic carbon of adenosine (m2A; Benitez-Paez et al., 2012). RlmN catalyzes the methylation reaction using a SAM radical rather than the usual nucleophilic substitution reaction mechanism (S_N_2 mechanism; Grove et al., 2011). The use of the SAM radical to perform an array of unusual and chemically difficult transformations is an ancient mechanism displayed by organisms grown anaerobically (Stubbe, 2011). These data suggest that RlmN is an ancient enzyme that appeared on Earth around 3 billion years ago when oxygen was not sufficiently abundant in the atmosphere (**Figure 3**).

## MTases with Both RNA- and DNA-Substrate Specificity

NSun2 and Dnmt2 are, at this time, the only known 5-methylcytidine (m5C) RNA MTases in higher eukaryotes; tRNA is the confirmed target substrate for both enzymes (Brzezicha et al., 2006; Goll et al., 2006). Recently, additional RNA substrates for NSun2 have been identified, including mRNAs and ncRNAs (Hussain et al., 2013). Furthermore, NSun2 deficiency has been linked to intellectual disability (Abbasi-Moheb et al., 2012). An interesting feature of NSun2 is its ability to methylate not only its RNA substrates, but also hemimethylated DNA (Sakita-Suto et al., 2007). Another example that supports the appearance during evolution of MTases with dual specificity for RNA and DNA is Dnmt2 (Hermann et al., 2003; Kunert et al., 2003; Jeltsch et al., 2006). Dnmt2 is spread throughout the eukaryotic kingdom from simple protists to complex metazoans (Schaefer and Lyko, 2009). Dnmt2-mediated tRNA methylation is associated with resistance to various stresses including heat shock, nitrosative stresses and oxidative stresses (Schaefer et al., 2010; Blanco et al., 2014; Hertz et al., 2014). Dnmt2 is primarily a tRNA MTase, but it employs a catalytic mechanism characteristic of a DNA-MTase (Jurkowski et al., 2008). According to a phylogenetic study, Dnmt2 has evolved from a DNA MTase ancestor and acquired the ability to methylate tRNA substrates (Jurkowski and Jeltsch, 2011). However, earlier bioinformatics and biochemical studies have suggested the opposite; Dnmt2 may have evolved from a hypothetical very ancient RNA: (pyrimidine, C5) methyltransferase (Anantharaman et al., 2002; Bujnicki et al., 2004; Sunita et al., 2008).

## Conserved Base Flipping Evolution from RNA to DNA

Base flipping is a biological process in which a specific nitrogenous base from the stacked region turns around 180° at the catalytic site of MTase without disturbing the remainder of the nucleic acid backbone (Roberts and Cheng, 1998). In the next step, the base is methylated and flipped back to its stacked conformation. The legacy of base flipping mechanism from the RNA world is evidenced by an ancient tRNA MTase, TrmL (spoUT family), which methylates in the loops and double-stranded regions of naked tRNAs (Alian et al., 2008; Hou and Perona, 2009; Christian et al., 2010; Hamdane et al., 2014). Base flipping is less pronounced with rRNAs which are usually in complexes with ribosomal proteins. This suggests that DNA MTases evolved their base flipping activity, which is probably the most necessary element of the methylation mechanism needed in the DNA world, from tRNA MTases.

## Phages Responsible for DNA-MTases Dissemination?

Bacterial genomes evolve rapidly through mutation, rearrangement and horizontal gene transfer (Juhas et al., 2009). Phages associated with these prokaryotes are thought to have evolved from cellular retrotransposons (Xiong and Eickbush, 1990) through gene shuﬄing (Powell et al., 2000) and are frequently involved in horizontal gene transfer (Kurland et al., 2003; McDaniel et al., 2010). They play an important role in enriching the bacterial genomes, for example, a prophage of the Wolbachia endosymbiont of the fruit fly encodes a DNA MTase (N6-adenine specific) which seems to have transferred this gene into the bacterial genome (α-proteobacteria; Saridaki et al., 2011). N6-adenine specific methylation at the origin of replication is directly linked with fast and faithful division of bacterial cells and so are their associated phages. Nevertheless, Rhizobium meliloti, Brucella abortus, Agrobacterium tumefaciens, Rhodobacter capsulatus and Caulobacter crescentus (all ∝-proteobacteria) contain N6-adenine DNA MTase gene intact in their genomes (Wright et al., 1997). The phenomenon of horizontal gene transfer from phages to bacteria may have contributed to the wide distribution of type II restriction as well as many DNA MTases in the prokaryotic system (Jeltsch and Pingoud, 1996). Both adenine- and cytosine-specific DNA MTases are known to exist in viruses and their homologous forms have been well established in bacteria (Schneider-Scherzer et al., 1990; Shields et al., 1990).

## Concluding Remarks and Perspective

The demonstration of RNA as an independent catalyst (Kruger et al., 1982) established it as the earliest genetic material in the prebiotic world before the appearance of DNA. The “RNA world” hypothesis is supported by many other independent lines of evidence, such as the observations that RNA is central to the translation process (Lodish et al., 2000), that some RNAs *in vitro* are capable of self-sustained replication (Lincoln and Joyce, 2009), that some can synthesize peptides (Hager et al., 1996) and that some RNAs (viroids; Flores et al., 2004) can catalyze all of the chemical group and information transfers required for cellular life. In the complexities of methylation reactions and MTases known today, we have selected here some unique MTases that provide an insight into how some ancient RNA-MTases evolved to methylate a different substrate. Many of these early MTases are multisite-specific tRNA MTases such as Trm4 and TrmH (Motorin and Grosjean, 1999; Auxilien et al., 2012; Ochi et al., 2013). Furthermore, some have a dual-substrate specificity, such as spoUT/RlmN (methylates rRNA as well as tRNA; Benitez-Paez et al., 2012) and NSUN2 (methylates both RNA and hemimethylated DNA; Sakita-Suto et al., 2007). It is tempting to speculate that this degree of flexibility for the substrate was an important feature of the early MTases which provided them the flexibility to switch from one substrate (RNA) to another (RNA/DNA). The dual-specificity spoUT family of MTases are a remarkable example of enzymes that thrived in the extreme conditions of the early Earth. Another MTase of the spoUT family, TrmL, demonstrates base flipping of its tRNA substrate during the methylation reaction similar to that observed in the DNA methylation mechanism, providing a possible RNA world-origin of this process which is necessary in the DNA world. Finally, we suggest that N6-adenine specific MTases, initially appeared as rRNA-MTases (Cotney and Shadel, 2006; Richter et al., 2009; Golovina et al., 2012) which evolved and adapted into DNA-MTases in the kingdom of eubacteria (Low et al., 2001) through rapid dispersal of DNA (horizontal transfers) possibly mediated through their phages.

The growing repertoire of RNA/DNA modifications dataset opens new avenues of research on the origin of nucleic acid modifications in the various kingdoms of life. This review has supplemented the concept of “RNA stability” to the “RNA world” hypothesis, which on evolution through the ages in the geological clock might have provided an avenue for the development of the more stable genetic material, “DNA.”

The appearance of replicating RNA/DNA, non-random genetic code, left-handed optical asymmetry of amino acids and right-handed sugar molecules could not be the mere result of chemicals laws, physical laws or laws of symmetry acting on the molecules in the primordial broth. In fact, these laws alone would have resulted in racemic mixtures incompatible with the evolution of current forms of life. The introduction of chirality in the early biomolecules was essential and formed the basis for the existence of life and it might be the effect of the early environment, climatic conditions and geomorphological history of the Earth that shaped the biomolecules into chiral (asymmetric) forms rather than racemic mixtures. Much remains to be understood about the exact nature of early Earth conditions, the constituents of the primordial mixture, the appearance of RNA and DNA, the introduction of chirality in shaping life and how methylation could be one of the mechanisms to conserve chirality in the biomolecules (Law and Jacobsen, 2010; Ichiyanagi et al., 2012).

## Author Contributions

All authors listed, have made substantial, direct and intellectual contribution to the work, and approved it for publication.

## Conflict of Interest Statement

The authors declare that the research was conducted in the absence of any commercial or financial relationships that could be construed as a potential conflict of interest.

## Footnotes

1 http://www.uniprot.org

## References

[B1] Abbasi-Moheb L. Mertel S. Gonsior M. Nouri-Vahid L. Kahrizi K. Cirak S. (2012). Mutations in NSUN2 cause autosomal-recessive intellectual disability. *Am. J. Hum. Genet.* 90 847–855. 10.1016/j.ajhg.2012.03.021 22541559 PMC3376487

[B2] Abouhaidar M. G. Ivanov I. G. (1999). Non-enzymatic RNA hydrolysis promoted by the combined catalytic activity of buffers and magnesium ions. *Z. Naturforsch. C* 54 542–548. 10.1515/znc-1999-7-813 10488562

[B3] Al-Arif A. Sporn M. B. (1972). 2’-O-methylation of adenosine, guanosine, uridine, and cytidine in RNA of isolated rat liver nuclei. *Proc. Natl. Acad. Sci. U.S.A.* 69 1716–1719. 10.1073/pnas.69.7.1716 4340155 PMC426785

[B4] Alian A. Lee T. T. Griner S. L. Stroud R. M. Finer-Moore J. (2008). Structure of a TrmA-RNA complex: a consensus RNA fold contributes to substrate selectivity and catalysis in m5U methyltransferases. *Proc. Natl. Acad. Sci. U.S.A.* 105 6876–6881. 10.1073/pnas.0802247105 18451029 PMC2383949

[B5] Anantharaman V. Koonin E. V. Aravind L. (2002). Comparative genomics and evolution of proteins involved in RNA metabolism. *Nucleic Acids Res.* 30 1427–1464. 10.1093/nar/30.7.1427 11917006 PMC101826

[B6] Attwater J. Wochner A. Holliger P. (2013). In-ice evolution of RNA polymerase ribozyme activity. *Nat. Chem.* 5 1011–1018. 10.1038/nchem.1781 24256864 PMC3920166

[B7] Auxilien S. Guerineau V. Szweykowska-Kulinska Z. Golinelli-Pimpaneau B. (2012). The human tRNA m (5) C methyltransferase Misu is multisite-specific. *RNA Biol.* 9 1331–1338. 10.4161/rna.22180 22995836 PMC3597573

[B8] Benitez-Paez A. Villarroya M. Armengod M. E. (2012). The *Escherichia coli* RlmN methyltransferase is a dual-specificity enzyme that modifies both rRNA and tRNA and controls translational accuracy. *RNA* 18 1783–1795. 10.1261/rna.033266.112 22891362 PMC3446703

[B9] Blanco S. Dietmann S. Flores J. V. Hussain S. Kutter C. Humphreys P. (2014). Aberrant methylation of tRNAs links cellular stress to neuro-developmental disorders. *EMBO J.* 33 2020–2039. 10.15252/embj.201489282 25063673 PMC4195770

[B10] Brzezicha B. Schmidt M. Makalowska I. Jarmolowski A. Pienkowska J. Szweykowska-Kulinska Z. (2006). Identification of human tRNA:m^5^C methyltransferase catalysing intron-dependent m^5^C formation in the first position of the anticodon of the ${\mathrm{pre-tRNA}}_{\mathrm{(CAA)}}^{Leu}$. *Nucleic Acids Res.* 34 6034–6043. 10.1093/nar/gkl765 17071714 PMC1635329

[B11] Bujnicki J. M. Feder M. Ayres C. L. Redman K. L. (2004). Sequence-structure-function studies of tRNA:m5C methyltransferase Trm4p and its relationship to DNA:m5C and RNA:m5U methyltransferases. *Nucleic Acids Res.* 32 2453–2463. 10.1093/nar/gkh564 15121902 PMC419452

[B12] Christian T. Lahoud G. Liu C. Hoffmann K. Perona J. J. Hou Y. M. (2010). Mechanism of N-methylation by the tRNA m1G37 methyltransferase Trm5. *RNA* 16 2484–2492. 10.1261/rna.2376210 20980671 PMC2995409

[B13] Clouet d’Orval B. Bortolin M. L. Gaspin C. Bachellerie J. P. (2001). Box C/D RNA guides for the ribose methylation of archaeal tRNAs. The tRNATrp intron guides the formation of two ribose-methylated nucleosides in the mature tRNATrp. *Nucleic Acids Res.* 29 4518–4529. 10.1093/nar/29.22.4518 11713301 PMC92551

[B14] Clouet-d’Orval B. Gaspin C. Mougin A. (2005). Two different mechanisms for tRNA ribose methylation in Archaea: a short survey. *Biochimie* 87 889–895. 10.1016/j.biochi.2005.02.004 16164996

[B15] Cooper G. M. (2000). *The Cell: A Molecular Approach I.The Origin and Evolution of Cells*. Sunderland, MA: Sinauer Associates.

[B16] Costanzo G. Saladino R. Crestini C. Ciciriello F. Di Mauro E. (2007). Nucleoside phosphorylation by phosphate minerals. *J. Biol. Chem.* 282 16729–16735. 10.1074/jbc.M611346200 17412692

[B17] Cotney J. Shadel G. S. (2006). Evidence for an early gene duplication event in the evolution of the mitochondrial transcription factor B family and maintenance of rRNA methyltransferase activity in human mtTFB1 and mtTFB2. *J. Mol. Evol.* 63 707–717. 10.1007/s00239-006-0075-1 17031457

[B18] Cui Z. Sun L. Zhang B. (2004). A peptidyl transferase ribozyme capable of combinatorial peptide synthesis. *Bioorg. Med. Chem.* 12 927–933. 10.1016/j.bmc.2003.12.018 14980605

[B19] Duthie S. J. Narayanan S. Brand G. M. Pirie L. Grant G. (2002). Impact of folate deficiency on DNA stability. *J. Nutr.* 132 2444S–2449S. 12163709 10.1093/jn/132.8.2444S

[B20] Ekland E. H. Bartel D. P. (1996). RNA-catalysed RNA polymerization using nucleoside triphosphates. *Nature* 382 373–376. 10.1038/382373a0 8684470

[B21] Feder M. Pas J. Wyrwicz L. S. Bujnicki J. M. (2003). Molecular phylogenetics of the RrmJ/fibrillarin superfamily of ribose 2’-O-methyltransferases. *Gene* 302 129–138. 10.1016/S0378-1119(02)01097-1 12527203

[B22] Ferre-D’Amare A. R. Scott W. G. (2010). Small self-cleaving ribozymes. *Cold Spring Harb. Perspect. Biol.* 2:a003574 10.1101/cshperspect.a003574 PMC2944367 20843979

[B23] Ferris J. P. Ertem G. (1993). Montmorillonite catalysis of RNA oligomer formation in aqueous solution. A model for the prebiotic formation of RNA. *J. Am. Chem. Soc.* 115 12270–12275. 10.1021/ja00079a006 11540110

[B24] Flores R. Delgado S. Gas M. E. Carbonell A. Molina D. Gago S. (2004). Viroids: the minimal non-coding RNAs with autonomous replication. *FEBS Lett.* 567 42–48. 10.1016/j.febslet.2004.03.118 15165891

[B26] Golovina A. Y. Dzama M. M. Osterman I. A. Sergiev P. V. Serebryakova M. V. Bogdanov A. A. (2012). The last rRNA methyltransferase of *E. coli* revealed: the yhiR gene encodes adenine-N6 methyltransferase specific for modification of A2030 of 23S ribosomal RNA. *RNA* 18 1725–1734. 10.1261/rna.034207.112 22847818 PMC3425786

[B27] Goll M. G. Kirpekar F. Maggert K. A. Yoder J. A. Hsieh C. L. Zhang X. (2006). Methylation of tRNA^Asp^ by the DNA methyltransferase homolog Dnmt2. *Science* 311 395–398. 10.1126/science.1120976 16424344

[B28] Goodchild J. (1992). Enhancement of ribozyme catalytic activity by a contiguous oligodeoxynucleotide (facilitator) and by 2’-O-methylation. *Nucleic Acids Res.* 20 4607–4612. 10.1093/nar/20.17.4607 1383929 PMC334191

[B29] Grove T. L. Benner J. S. Radle M. I. Ahlum J. H. Landgraf B. J. Krebs C. (2011). A radically different mechanism for S-adenosylmethionine-dependent methyltransferases. *Science* 332 604–607. 10.1126/science.1200877 21415317

[B30] Hager A. J. Pollard J. D. Szostak J. W. (1996). Ribozymes: aiming at RNA replication and protein synthesis. *Chem. Biol.* 3 717–725. 10.1016/S1074-5521(96)90246-X 8939686

[B31] Hamdane D. Guelorget A. Guerineau V. Golinelli-Pimpaneau B. (2014). Dynamics of RNA modification by a multi-site-specific tRNA methyltransferase. *Nucleic Acids Res.* 42 11697–11706. 10.1093/nar/gku820 25217588 PMC4191401

[B32] Harish A. Caetano-Anolles G. (2012). Ribosomal history reveals origins of modern protein synthesis. *PLoS ONE* 7:e32776 10.1371/journal.pone.0032776 PMC3299690 22427882

[B33] Hermann A. Schmitt S. Jeltsch A. (2003). The human Dnmt2 has residual DNA-(cytosine-C5) methyltransferase activity. *J. Biol. Chem.* 278 31717–31721. 10.1074/jbc.M305448200 12794065

[B34] Hertz R. Tovy A. Kirschenbaum M. Geffen M. Nozaki T. Adir N. (2014). The *Entamoeba histolytica* Dnmt2 homolog (Ehmeth) confers resistance to nitrosative stress. *Eukaryot. Cell* 13 494–503. 10.1128/EC.00031-14 24562908 PMC4000097

[B35] Higgs P. G. Lehman N. (2015). The RNA World: molecular cooperation at the origins of life. *Nat. Rev. Genet.* 16 7–17. 10.1038/nrg3841 25385129

[B36] Hirao I. Ellington A. D. (1995). Re-creating the RNA world. *Curr. Biol.* 5 1017–1022. 10.1016/S0960-9822(95)00205-3 8542277

[B37] Hou Y. M. Perona J. J. (2009). Stereochemical mechanisms of tRNA methyltransferases. *FEBS Lett.* 584 278–286. 10.1016/j.febslet.2009.11.075 19944101 PMC2797553

[B38] Hussain S. Sajini A. A. Blanco S. Dietmann S. Lombard P. Sugimoto Y. (2013). NSun2-mediated cytosine-5 methylation of vault noncoding RNA determines its processing into regulatory small RNAs. *Cell Rep.* 4 255–261. 10.1016/j.celrep.2013.06.029 23871666 PMC3730056

[B39] Ichiyanagi T. Ichiyanagi K. Miyake M. Sasaki H. (2012). Accumulation and loss of asymmetric non-CpG methylation during male germ-cell development. *Nucleic Acids Res.* 41 738–745. 10.1093/nar/gks1117 23180759 PMC3553940

[B40] Jayasena V. K. Gold L. (1997). In vitro selection of self-cleaving RNAs with a low pH optimum. *Proc. Natl. Acad. Sci. U.S.A.* 94 10612–10617. 10.1073/pnas.94.20.10612 9380683 PMC23421

[B41] Jeffares D. C. Poole A. M. Penny D. (1995). Pre-rRNA processing and the path from the RNA world. *Trends Biochem. Sci.* 20 298–299. 10.1016/S0968-0004(00)89053-2 7545338

[B42] Jeltsch A. Nellen W. Lyko F. (2006). Two substrates are better than one: dual specificities for Dnmt2 methyltransferases. *Trends Biochem. Sci.* 31 306–308. 10.1016/j.tibs.2006.04.005 16679017

[B43] Jeltsch A. Pingoud A. (1996). Horizontal gene transfer contributes to the wide distribution and evolution of type II restriction-modification systems. *J. Mol. Evol.* 42 91–96. 10.1007/BF02198833 8919860

[B44] Jeruzalmi D. Steitz T. A. (1998). Structure of T7 RNA polymerase complexed to the transcriptional inhibitor T7 lysozyme. *EMBO J.* 17 4101–4113. 10.1093/emboj/17.14.4101 9670025 PMC1170743

[B45] Johnston W. K. Unrau P. J. Lawrence M. S. Glasner M. E. Bartel D. P. (2001). RNA-catalyzed RNA polymerization: accurate and general RNA-templated primer extension. *Science* 292 1319–1325. 10.1126/science.1060786 11358999

[B46] Juhas M. Van Der Meer J. R. Gaillard M. Harding R. M. Hood D. W. Crook D. W. (2009). Genomic islands: tools of bacterial horizontal gene transfer and evolution. *FEMS Microbiol. Rev.* 33 376–393. 10.1111/j.1574-6976.2008.00136.x 19178566 PMC2704930

[B47] Jurkowski T. P. Jeltsch A. (2011). On the evolutionary origin of eukaryotic DNA methyltransferases and Dnmt2. *PLoS ONE* 6:e28104 10.1371/journal.pone.0028104 PMC3227630 22140515

[B48] Jurkowski T. P. Meusburger M. Phalke S. Helm M. Nellen W. Reuter G. (2008). Human DNMT2 methylates tRNA(Asp) molecules using a DNA methyltransferase-like catalytic mechanism. *RNA* 14 1663–1670. 10.1261/rna.970408 18567810 PMC2491481

[B49] Keefe A. D. Miller S. L. Mcdonald G. Bada J. (1995). Investigation of the prebiotic synthesis of amino acids and RNA bases from CO_2_ using FeS/H2S as a reducing agent. *Proc. Natl. Acad. Sci. U.S.A.* 92 11904–11906. 10.1073/pnas.92.25.11904 8524872 PMC40511

[B50] Kikovska E. Mikkelsen N. E. Kirsebom L. A. (2005). The naturally trans-acting ribozyme RNase P RNA has leadzyme properties. *Nucleic Acids Res.* 33 6920–6930. 10.1093/nar/gki993 16332695 PMC1310964

[B51] Kolb V. M. Dworkin J. P. Miller S. L. (1994). Alternative bases in the RNA world: the prebiotic synthesis of urazole and its ribosides. *J. Mol. Evol.* 38 549–557. 10.1007/BF00175873 11539446

[B52] Koonin E. V. Dolja V. V. (2014). Virus world as an evolutionary network of viruses and capsidless selfish elements. *Microbiol. Mol. Biol. Rev.* 78 278–303. 10.1128/MMBR.00049-13 24847023 PMC4054253

[B53] Kruger K. Grabowski P. J. Zaug A. J. Sands J. Gottschling D. E. Cech T. R. (1982). Self-splicing RNA: autoexcision and autocyclization of the ribosomal RNA intervening sequence of *Tetrahymena*. *Cell* 31 147–157. 10.1016/0092-8674(82)90414-7 6297745

[B54] Kunert N. Marhold J. Stanke J. Stach D. Lyko F. (2003). A Dnmt2-like protein mediates DNA methylation in *Drosophila*. *Development* 130 5083–5090. 10.1242/dev.00716 12944428

[B55] Kurland C. G. Canback B. Berg O. G. (2003). Horizontal gene transfer: a critical view. *Proc. Natl. Acad. Sci. U.S.A.* 100 9658–9662. 10.1073/pnas.1632870100 12902542 PMC187805

[B56] Law J. A. Jacobsen S. E. (2010). Establishing, maintaining and modifying DNA methylation patterns in plants and animals. *Nat. Rev. Genet.* 11 204–220. 10.1038/nrg2719 20142834 PMC3034103

[B57] Levy M. Miller S. L. (1998). The stability of the RNA bases: implications for the origin of life. *Proc. Natl. Acad. Sci. U.S.A.* 95 7933–7938. 10.1073/pnas.95.14.7933 9653118 PMC20907

[B58] Lincoln T. A. Joyce G. F. (2009). Self-sustained replication of an RNA enzyme. *Science* 323 1229–1232. 10.1126/science.1167856 19131595 PMC2652413

[B59] Liu R. J. Zhou M. Fang Z. P. Wang M. Zhou X. L. Wang E. D. (2013). The tRNA recognition mechanism of the minimalist SPOUT methyltransferase, TrmL. *Nucleic Acids Res.* 41 7828–7842. 10.1093/nar/gkt568 23804755 PMC3763551

[B60] Lodish H. Berk A. Zipursky S. (2000). *The Three Roles of RNA in Protein Synthesis.* New York, NY: W. H. Freeman.

[B61] Long D. M. Uhlenbeck O. C. (1993). Self-cleaving catalytic RNA. *FASEB J.* 7 25–30. 8422971 10.1096/fasebj.7.1.8422971

[B62] Low D. A. Weyand N. J. Mahan M. J. (2001). Roles of DNA adenine methylation in regulating bacterial gene expression and virulence. *Infect. Immun.* 69 7197–7204. 10.1128/IAI.69.12.7197-7204.2001 11705888 PMC98802

[B63] Manrubia S. C. Briones C. (2007). Modular evolution and increase of functional complexity in replicating RNA molecules. *RNA* 13 97–107. 10.1261/rna.203006 17105993 PMC1705761

[B64] McDaniel L. D. Young E. Delaney J. Ruhnau F. Ritchie K. B. Paul J. H. (2010). High frequency of horizontal gene transfer in the oceans. *Science* 330:50 10.1126/science.1192243 20929803

[B65] Melendez-Hevia E. (2009). From the RNA world to the DNA-protein world: clues to the origin and early evolution of life in the ribosome. *J. Biosci.* 34 825–827. 10.1007/s12038-009-0095-2 20093734

[B66] Meyers L. A. Lee J. F. Cowperthwaite M. Ellington A. D. (2004). The robustness of naturally and artificially selected nucleic acid secondary structures. *J. Mol. Evol.* 58 681–691. 10.1007/s00239-004-2590-2 15461425

[B67] Mihalusova M. Wu J. Y. Zhuang X. (2011). Functional importance of telomerase pseudoknot revealed by single-molecule analysis. *Proc. Natl. Acad. Sci. U.S.A.* 108 20339–20344. 10.1073/pnas.1017686108 21571642 PMC3251044

[B68] Miller S. L. (1953). A production of amino acids under possible primitive Earth conditions. *Science* 117 528–529. 10.1126/science.117.3046.528 13056598

[B69] Miller S. L. Lazcano A. (1995). The origin of life–did it occur at high temperatures? *J. Mol. Evol.* 41 689–692. 10.1007/BF00173146 11539558

[B70] Miller S. L. Schlesinger G. (1983). The atmosphere of the primitive Earth and the prebiotic synthesis of organic compounds. *Adv. Space Res.* 3 47–53. 10.1016/0273-1177(83)90040-6 11542461

[B71] Miller S. L. Urey H. C. (1959). Organic compound synthesis on the primitive Earth. *Science* 130 245–251. 10.1126/science.130.3370.245 13668555

[B72] Mishanina T. V. Koehn E. M. Kohen A. (2012). Mechanisms and inhibition of uracil methylating enzymes. *Bioorg. Chem.* 43 37–43. 10.1016/j.bioorg.2011.11.005 22172597 PMC3315608

[B73] Motorin Y. Grosjean H. (1999). Multisite-specific tRNA:m5C-methyltransferase (Trm4) in yeast *Saccharomyces cerevisiae*: identification of the gene and substrate specificity of the enzyme. *RNA* 5 1105–1118. 10.1017/S1355838299982201 10445884 PMC1369833

[B74] Muller U. F. (2006). Re-creating an RNA world. *Cell Mol. Life. Sci.* 63 1278–1293. 10.1007/s00018-006-6047-1 16649141 PMC11136017

[B75] Ochi A. Makabe K. Yamagami R. Hirata A. Sakaguchi R. Hou Y. M. (2013). The catalytic domain of topological knot tRNA methyltransferase (TrmH) discriminates between substrate tRNA and nonsubstrate tRNA via an induced-fit process. *J. Biol. Chem.* 288 25562–25574. 10.1074/jbc.M113.485128 23867454 PMC3757217

[B76] Orgel L. E. (2004). Prebiotic chemistry and the origin of the RNA world. *Crit. Rev. Biochem. Mol. Biol.* 39 99–123. 10.1080/10409230490460765 15217990

[B77] Paolella G. Sproat B. S. Lamond A. I. (1992). Nuclease resistant ribozymes with high catalytic activity. *EMBO J.* 11 1913–1919. 1582419 10.1002/j.1460-2075.1992.tb05244.x PMC556650

[B78] Parker E. T. Cleaves H. J. Callahan M. P. Dworkin J. P. Glavin D. P. Lazcano A. (2011). Prebiotic synthesis of methionine and other sulfur-containing organic compounds on the primitive Earth: a contemporary reassessment based on an unpublished 1958 Stanley Miller experiment. *Orig. Life Evol. Biosph.* 41 201–212. 10.1007/s11084-010-9228-8 21063908 PMC3094541

[B79] Poole A. Penny D. Sjoberg B. (2000). Methyl-RNA: an evolutionary bridge between RNA and DNA? *Chem. Biol.* 7 R207–R216. 10.1016/S1074-5521(00)00042-9 11137821 PMC7172353

[B80] Poole A. M. Jeffares D. C. Penny D. (1998). The path from the RNA world. *J. Mol. Evol.* 46 1–17. 10.1007/PL00006275 9419221

[B81] Powell S. K. Kaloss M. A. Pinkstaff A. Mckee R. Burimski I. Pensiero M. (2000). Breeding of retroviruses by DNA shuﬄing for improved stability and processing yields. *Nat. Biotechnol.* 18 1279–1282. 10.1038/82391 11101807

[B82] Pressman A. Blanco C. Chen I. A. (2015). The RNA world as a model system to study the origin of life. *Curr. Biol.* 25 R953–R963. 10.1016/j.cub.2015.06.016 26439358

[B83] Reichard P. (1993). From RNA to DNA, why so many ribonucleotide reductases? *Science* 260 1773–1777. 10.1126/science.8511586 8511586

[B84] Richter U. Kuhn K. Okada S. Brennicke A. Weihe A. Borner T. (2009). A mitochondrial rRNA dimethyladenosine methyltransferase in *Arabidopsis*. *Plant J.* 61 558–569. 10.1111/j.1365-313X.2009.04079.x 19929881 PMC2860759

[B85] Rios A. C. Yu H. T. Tor Y. (2014). Hydrolytic fitness of N-glycosyl bonds: comparing the deglycosylation kinetics of modified, alternative, and native nucleosides. *J. Phys. Org. Chem.* 28 173–180. 10.1002/poc.3318 25750482 PMC4349208

[B86] Roberts R. J. Cheng X. (1998). Base flipping. *Annu. Rev. Biochem.* 67 181–198. 10.1146/annurev.biochem.67.1.181 9759487

[B87] Robertson M. P. Miller S. L. (1995). An efficient prebiotic synthesis of cytosine and uracil. *Nature* 375 772–774. 10.1038/375772a0 7596408

[B88] Sakita-Suto S. Kanda A. Suzuki F. Sato S. Takata T. Tatsuka M. (2007). Aurora-B regulates RNA methyltransferase NSUN2. *Mol. Biol. Cell* 18 1107–1117. 10.1091/mbc.E06-11-1021 17215513 PMC1805108

[B89] Saridaki A. Sapountzis P. Harris H. L. Batista P. D. Biliske J. A. Pavlikaki H. (2011). Wolbachia prophage DNA adenine methyltransferase genes in different *Drosophila*-Wolbachia associations. *PLoS ONE* 6:e19708 10.1371/journal.pone.0019708 PMC3089641 21573076

[B90] Schaefer M. Lyko F. (2009). Solving the Dnmt2 enigma. *Chromosoma* 119 35–40. 10.1007/s00412-009-0240-6 19730874

[B91] Schaefer M. Pollex T. Hanna K. Tuorto F. Meusburger M. Helm M. (2010). RNA methylation by Dnmt2 protects transfer RNAs against stress-induced cleavage. *Genes Dev.* 24 1590–1595. 10.1101/gad.586710 20679393 PMC2912555

[B92] Schimmel P. Henderson B. (1994). Possible role of aminoacyl-RNA complexes in noncoded peptide synthesis and origin of coded synthesis. *Proc. Natl. Acad. Sci. U.S.A.* 91 11283–11286. 10.1073/pnas.91.24.11283 7972050 PMC45215

[B93] Schneider-Scherzer E. Auer B. De Groot E. J. Schweiger M. (1990). Primary structure of a DNA (N6-adenine)-methyltransferase from *Escherichia coli* virus T1. DNA sequence, genomic organization, and comparative analysis. *J. Biol. Chem.* 265 6086–6091. 2180941

[B94] Schrader M. E. (2009). The RNA world: conditions for prebiotic synthesis. *J. Geophys. Res.* 114 D15305–D15311. 10.1029/2008JD010810

[B95] Shapiro R. (1988). Prebiotic ribose synthesis: a critical analysis. *Orig. Life Evol. Biosph.* 18 71–85. 10.1007/BF01808782 2453009

[B96] Shechner D. M. Bartel D. P. (2011). The structural basis of RNA-catalyzed RNA polymerization. *Nat. Struct. Mol. Biol.* 18 1036–1042. 10.1038/nsmb.2107 21857665 PMC3169305

[B97] Shields S. L. Burbank D. E. Grabherr R. Van Etten J. L. (1990). Cloning and sequencing the cytosine methyltransferase gene M. CviJI from Chlorella virus IL-3A. *Virology* 176 16–24. 2158687 10.1016/0042-6822(90)90225-g

[B98] Steitz T. A. Steitz J. A. (1993). A general two-metal-ion mechanism for catalytic RNA. *Proc. Natl. Acad. Sci. U.S.A.* 90 6498–6502. 10.1073/pnas.90.14.6498 8341661 PMC46959

[B99] Stubbe J. (2011). Biochemistry. The two faces of SAM. *Science* 332 544–545. 21527702 10.1126/science.1204209

[B100] Sun L. Cui Z. Gottlieb R. L. Zhang B. (2002). A selected ribozyme catalyzing diverse dipeptide synthesis. *Chem. Biol.* 9 619–628. 10.1016/S1074-5521(02)00141-2 12031668

[B101] Sunita S. Tkaczuk K. L. Purta E. Kasprzak J. M. Douthwaite S. Bujnicki J. M. (2008). Crystal structure of the *Escherichia coli* 23S rRNA:m5C methyltransferase RlmI (YccW) reveals evolutionary links between RNA modification enzymes. *J. Mol. Biol.* 383 652–666. 10.1016/j.jmb.2008.08.062 18789337

[B102] Tamura K. Schimmel P. (2001). Oligonucleotide-directed peptide synthesis in a ribosome- and ribozyme-free system. *Proc. Natl. Acad. Sci. U.S.A.* 98 1393–1397. 10.1073/pnas.98.4.1393 11171961 PMC29267

[B103] Tamura K. Schimmel P. (2003). Peptide synthesis with a template-like RNA guide and aminoacyl phosphate adaptors. *Proc. Natl. Acad. Sci. U.S.A.* 100 8666–8669. 10.1073/pnas.1432909100 12857953 PMC166369

[B104] Tsukada Y. (2012). Hydroxylation mediates chromatin demethylation. *J. Biochem.* 151 229–246. 10.1093/jb/mvs003 22247561

[B105] Usher D. A. McHale A. H. (1976). Hydrolytic stability of helical RNA: a selective advantage for the natural 3’,5’-bond. *Proc. Natl. Acad. Sci. U.S.A.* 73 1149–1153. 10.1073/pnas.73.4.1149 1063396 PMC430218

[B106] Van Trump J. E. Miller S. L. (1972). Prebiotic synthesis of methionine. *Science* 178 859–860. 10.1126/science.178.4063.859 5085982

[B107] Wright R. Stephens C. Shapiro L. (1997). The CcrM DNA methyltransferase is widespread in the alpha subdivision of proteobacteria, and its essential functions are conserved in *Rhizobium meliloti* and *Caulobacter crescentus*. *J. Bacteriol.* 179 5869–5877. 9294447 10.1128/jb.179.18.5869-5877.1997 PMC179479

[B108] Xiong Y. Eickbush T. H. (1990). Origin and evolution of retroelements based upon their reverse transcriptase sequences. *EMBO J.* 9 3353–3362. 1698615 10.1002/j.1460-2075.1990.tb07536.x PMC552073

[B109] Zaher H. S. Unrau P. J. (2007). Selection of an improved RNA polymerase ribozyme with superior extension and fidelity. *RNA* 13 1017–1026. 10.1261/rna.548807 17586759 PMC1894930

[B110] Zenkin N. (2012). Hypothesis: emergence of translation as a result of RNA helicase evolution. *J. Mol. Evol.* 74 249–256. 10.1007/s00239-012-9503-6 22544085

[B111] Zhang Q. Van Der Donk W. A. Liu W. (2011). Radical-mediated enzymatic methylation: a tale of two SAMS. *Acc. Chem. Res.* 45 555–564. 10.1021/ar200202c 22097883 PMC3328197

